# Evaluation of the Use of Anticoagulotherapy in Cancer Patients in Palliative Care Residence

**DOI:** 10.1089/pmr.2022.0069

**Published:** 2023-02-20

**Authors:** Stefano Polesello, Sebastian Georgescu, Talía Malagón, Sylvie Bouchard

**Affiliations:** ^1^ Department of Palliative Medicine, McGill University, Montreal, Quebec, Canada.; ^2^ Department of Oncology, Division of Cancer Epidemiology, McGill University, Montreal, Quebec, Canada.; ^3^ Montreal Institute for Palliative Care/Teresa Dellar Palliative Care Residence, Department of Oncology, McGill University, Montreal, Quebec, Canada.

**Keywords:** anticoagulation, bleeding, cancer, palliative care, hospice, pulmonary embolism, venous thromboembolism

## Abstract

**Background:**

Several patients admitted to palliative care residences are on anticoagulotherapy (AC). Given the risks of venous thromboembolism (VTE) and bleeding, the decision to continue or stop AC on admission remains clinically challenging.

**Objectives:**

To determine the prevalence of AC use and incidence of suspected VTE and bleeding events in palliative care patients.

**Methods:**

Retrospective cohort study including all deceased patients at a Canadian palliative care residence over two years.

**Results:**

Among the 453 patients' charts reviewed (369 with cancer), 183 (40%) were on AC at admission or <30 days earlier. Only 64 (35%) continued AC, with 78% discontinuing it during their stay. Demographic parameters were similar in the AC and non-AC groups. The incidence of suspected VTE was lower in patients pursuing AC post-admission than in those who stopped: (4.6% vs. 6.7%) and, conversely, the incidence of bleeding was higher in patients on AC: (10.8% vs. 7.6%), though these differences were not statistically significant. The risk of death in cancer patients within 72 hours of suspected VTE or bleeding event was 80% and 30%, respectively. Patients on AC had a 33% reduced risk of VTE but a 44% increased risk of bleeding.

**Conclusion:**

This study provides information on the AC use in palliative care patients. In term of survivorship, it suggests a possible advantage to continue AC to prevent a symptomatic or distressing death. Given the low incidence of events, larger powered studies will be necessary to further characterize the risks/benefits of pursuing AC in patients in palliative care residences.

## Introduction

It has been well established that patients with decreased mobility, advanced age, or active cancer are at high risk of developing venous thromboembolism (VTE). Thromboprophylaxis (TPX) is currently used as standard practice in these patients during hospitalization.^1^

However, when life expectancy of this patient population becomes limited, such as in those admitted to hospices for end-of-life care, the decision to continue TPX remains a challenge. Indeed, there is little evidence to strongly support the continuation or cessation of TPX since clinical trials investigating VTE prophylaxis in the population of cancer patients usually exclude palliative care patients.^2^ In addition, patient factors and comorbidities seem to not be strongly associated with the decision to discontinue TPX.^3^ Due to these factors, most clinicians rely on their own assessment and experience.

Previous studies have demonstrated a significantly high incidence of VTE in the palliative population among hospices. A study by Ambrus et al reported hemorrhagic and thromboembolic phenomena as contributory causes of death in over 40% of cancer patients.^4^ Using light reflection rheography for screening, Johnson et al. found a prevalence of 50% for deep vein thrombosis (DVT) in a sample of 98 cancer patients.^5^ Although there would be evidence to show this high prevalence of VTE in a hospice palliative care population, the question of preventable events or symptomatic benefit is an entirely different one.

More recently, one study established that although there was a significantly high burden of DVT with initial screening on arrival to a palliative care unit (27%), there was minimal symptomatic difference between those with and without DVT, in addition to no change in survival.^6^ A large multi-center observational study of 1191 patients in palliative care units also showed a very low incidence of DVT (0.5%) compared with higher (9.8%) prevalence of clinically significant bleeding.^7^

The actual burden of VTE in patients remains elusive. In a case series by Noble et al,^8^ no change in symptoms attributable to VTE was revealed between groups of patients who either continued their TPX until death, stopped up to one week before death, between one week and one month before death, or over one month before death. However, around 7% of patients who continued TPX until death or up to one week before death experienced clinically relevant non-major bleeding events.^8^

Bleeding is a clinically important adverse event in patients who are undergoing antithrombotic therapy. It is distressing to the patient, their family and is potentially life-threatening. Abnormal hepatic or renal function, metastatic disease, malnutrition, and immobility are known to be risk factors for fatal bleeding. Considering that hospice patients commonly have a combination of these factors, risk of bleeding while on antithrombotic therapy in this subpopulation may be even higher.

In the only randomized study to date looking at the palliative inpatient population, 20 patients with advanced cancer were administered either TPX daily or no treatment. In the group receiving TPX, one VTE and one major bleeding occurred, whereas two minor bleedings occurred in the control group: No statistically significant difference between the two groups was found.

No clear conclusion was established in terms of benefits or obvious disadvantages for primary TPX.^9^ In the previously mentioned RHESO study, the cumulative bleeding incidence at three months among all patients was 9.8%, much higher than the ∼1%–3% reported in acutely ill, bedridden patients. Interestingly, bleeding occurred in 11.0% of patients who received TPX and in 8.4% of those who did not. TPX was determined to be a significant independent risk factor for bleeding.^7^

These studies so far have pointed at the paucity of official guidelines and the difficulty in establishing clear guidelines for the use of anticoagulotherapy (AC) in the palliative hospice patient population.^10^ There is a challenging risk-benefit debate between the alleged benefit of reducing potentially symptomatic VTE, whereas there is a clear and possibly increased risk of bleeding in the same population.

In this study, the general use of AC within a Canadian hospice palliative care population, mostly with cancer, was described. In addition, the clinically apparent incidences of suspected VTE, or bleeding events were retrospectively observed.

## Materials and Methods

### Study design

This was a retrospective cohort study observing the outcome of deceased patients at the Teresa Dellar Palliative Care Residence, during a period of two years. Ethical approval was received from the Faculty of Medicine McGill Institutional Review Board. No patient consent was required given that the data were limited to deceased patients. To maintain individual patients' confidentiality, the data were recorded anonymously, on an electronic spreadsheet from patients' electronic records (Medesync) at the Residence.

The available information in the selected period charts was reviewed to document a detailed history of the use or discontinuation of AC for VTE and non-VTE indications and clinical information relevant to suspected or diagnosed VTE or bleeding events. All cancer patients admitted to hospice had stopped cancer treatments.

### Event definitions

Given the retrospective nature of this study and the absence of radiological tools available in a palliative care setting, the events of interest (VTE, bleeding) were assessed clinically only, hence the use of the term “suspected” events. The clinical criteria used for suspected VTE were:

- For DVT: sudden leg pain with at least one other supporting clinical feature (such as asymmetrical swelling).
- For pulmonary embolism: The patient was documented with sudden and distressing dyspnea and/or chest pain with a deviation from the patient's expected clinical course (such as a significant decline in function, a sudden and persistent increase in respiratory symptoms).

Bleeding assessment was based on overt signs of bleeding such as hematuria, epistaxis, and gastro-intestinal bleed documented in the chart by nurses and/or physicians.

### Inclusion/exclusion criteria

All deceased patients admitted at the palliative care residence from January 1st 2020 to December 31st 2021 were included. Patients who left the premises for reasons other than death (i.e., relocation) were not included in this study. There were no other exclusion criteria.

### Primary endpoints

Primary endpoints consisted of:

- determining the incidence of suspected VTE and bleeding events after admission to the palliative care Residence,
- evaluating the prevalence of AC use in all patients before, at or post-admission at the Residence, to determine current clinical practices in terms of the continuation of AC on admission, and,
- evaluating the association between the bleeding or suspected VTE occurrence and the use or cessation of AC.

### Secondary endpoint

Evaluation of the proportion of patients experiencing death within 72 hours after the occurrence of a bleeding event or suspected VTE.

## Data Handling and Statistical Methods

Data handling and statistical analyses were performed using SAS 9.4 and R statistical software.

Sample size was based on feasibility (time allocated to the project during fellowship). The total population was stratified by cancer and non-cancer diagnosis. Demographic and other baseline characteristics were summarized, and descriptive statistics were performed for each group. Comorbidities were not included in the dataset.

Data was obtained for all patients to assess baseline characteristics of patient subgroups and verify whether there were meaningful differences acting as potential confounders. Use of AC was divided into three subgroups, depending on whether:

- the patient had been on AC within the past 30 days before admission,
- AC was continued at admission, and
- AC was stopped or not during the stay at the Residence.

Finally, the chart was reviewed to assess for clinical evidence of bleeding events or VTE.

### Analysis of primary endpoints

The cumulative incidence of suspected VTE or bleeding events and their association with use or cessation of AC was assessed using cumulative incidence functions, which estimate the marginal probability for each event in the presence of the competing risk of death. Gray's non-parametric test^10^ was used for equality of cumulative incidence functions as well as Fine and Gray's proportional hazards model^11^ to assess differences in risk of thrombotic or bleeding events between patients stratified by AC use.

AC was analyzed both as a time-fixed risk factor at admission (any AC use after admission vs. no AC use after admission) and as a time-varying risk factor (patients considered exposed while on AC, but unexposed after cessation of AC or if they were never on AC) in separate models. We fitted both univariate models to assess the crude association with AC, and models adjusted for potential confounders such as age, sex, cancer, or other pathologies.

Competing risk analyses were selected rather than product limit analyses because death is an important competing risk in this study, and we were interested in the marginal probabilities of experiencing thrombotic or bleeding events in this cohort.

### Analysis of secondary endpoints

Binomial exact 95% confidence intervals (CIs) were calculated for the proportion of patients experiencing death within 72 hours after experiencing a suspected thrombotic or bleeding event.

## Results

### Patient characteristics

A total of 453 patients' data met the inclusion criteria. Patient demographics were similar between the two groups: taking AC or not during their stay at the Residence (Table 1). Median age was 80 years, with 45% males and 55% females. The median time to death from admission was 10 days. Most patients had cancer (81%), with lung and colorectal cancers being the most prevalent (18% and 10% respectively).

**Table 1.** Characteristics of Study Population

Characteristic	No AC use <30 days before admission, *n* (%)	AC use stopped at admission, *n* (%)	Any AC use during stay, *n* (%)	Total, *n* (%)
Age (years), median (IQR)	81 (70–88)	80 (72–88)	81 (74–85)	80 (70–88)
Time to death from admission (days), median (IQR)	11 (4–24)	8 (3–18)	12 (6–27)	10 (4–22)
Total	269 (59)	119 (26)	65 (14.0)	453 (100.0)
Sex				
Male	130 (48.0)	46 (39.0)	28 (43.0)	204 (45.0)
Female	139 (52.0)	73 (61.0)	37 (57.0)	249 (55.0)
Cancer				
No	58 (22.0)	19 (16.0)	7 (11.0)	84 (19.0)
Yes	211 (78.0)	100 (84.0)	58 (89.0)	369 (81.0)
Cancer type				
Breast	14 (5.0)	13 (11.0)	4 (6.0)	31 (7.0)
Colorectal	29 (11.0)	13 (11.0)	5 (8.0)	47 (10.0)
Lung	39 (15.0)	27 (23.0)	15 (23.0)	81 (18.0)
Pancreatic	14 (5.0)	8 (7.0)	7 (11.0)	29 (6.0)
Prostate	16 (6.0)	6 (5.0)	3 (5.0)	25 (6.0)
Other	99 (37.0)	33 (27.7)	24 (40.0)	156 (34.4)
History before admission				
VTE	24 (9.0)	29 (24.0)	35 (54.0)	88 (19.0)
Bleeding	34 (13.0)	12 (10.0)	2 (3.0)	48 (11.0)
Both	6 (2.0)	12 (10.0)	8 (12.0)	26 (6.0)
Neither	205 (76.0)	66 (55.0)	20 (31.0)	291 (64.0)
AC use <30 days before admission				
Yes	0	119 (100.0)	64 (98.0)	183 (40.0)
No	269 (100.0)	0	1 (2.0)	270 (60.0)
AC type (before admission)				
Apixaban	5 (2.0)	17 (14.0)	33 (51.0)	55 (12.0)
Dalteparin	1 (0.4)	36 (30.0)	17 (26.0)	54 (12.0)
Others	5 (2.0)	101 (85.0)	32 (49.0)	138 (31.0)
None	259 (96.0)	1 (1.0)	0	260 (57.0)

AC, anticoagulotherapy; IQR, interquartile range; VTE, venous thromboembolism.

Most patients who continued taking AC during their stay had a previous history of VTE (54%). Only a few patients who had experienced bleeding before admission were administered AC during their stay at the Residence (3%). The most common anticoagulants used were Apixaban (51%) and Dalteparin (26%).

### Prevalence of AC use

Fourteen percent (65/453) of patients received AC at any point during their stay at the Residence. The decision regarding the continuation of AC at admission is presented in Table 2 for the 183 patients (157 with cancer) who were on AC within the 30 days before admission. Only 35% of patients (64/183) continued AC after admission, with the others stopping before or at admission. For the cancer subgroup, the same proportion of patients (36%; 57/157) continued receiving AC.

**Table 2.** Decision Regarding Continuation of Anticoagulotherapy USE at Admission in 183 Patients Who Were on Anticoagulotherapy During the <30 Days Before Admission^*^

Decision at admission	All *n* (%)	Cancer *n* (%)	Non-cancer *n* (%)
Continue AC but stopped prior to death	64 (35) 50 (78)	57 (36) 45 (79)	7 (27) 5 (71)
Stop AC at admission	54 (30)	47 (30)	7 (27)
Stopped AC prior to admission	65 (36)	53 (34)	12 (46)
Total	183	157	26

^*^ One patient started taking AC after admission.

The AC administration was stopped in most patients (78%; 50/64) during their stay at the Residence. Among the patients not taking AC within the 30 days before admission, only one was started on AC.

### Incidence of suspected VTE and bleeding

**FIG. 1.**
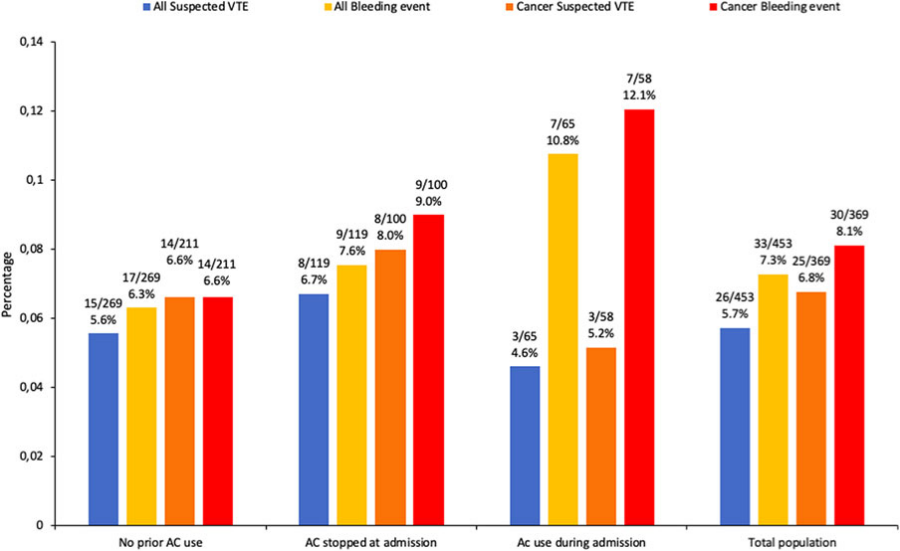
Probability of suspected thrombotic and bleeding events during palliative care stay at the Residence. AC, anticoagulotherapy; VTE, venous thromboembolism.

The cumulative probability of experiencing a possible VTE or bleeding event during the palliative care stay was 5.7% (26/453) and 7.3% (33/453), respectively (Fig. 1; total population). The probability of VTE was lower in patients who continued AC after admission (4.6%; 3/65) than in those who stopped AC at admission (6.7%; 8/119).

However, the risk of VTE was not significantly different between patients with any AC use after admission and those not on AC after admission (hazard ratio [HR] 0.66 [95% CI 0.18–2.47]) (Table 3). The probability of bleeding was higher in patients on AC during their stay (10.8%; 7/65) than in those who had their AC stopped at admission (7.6%; 9/119). However, the risk of bleeding was not significantly different between patients with any AC use after admission compared with those not on AC after admission (HR 1.44 [95% CI 0.54–3.85]).

**Table 3.** Hazard Ratio of Occurrence of Thrombotic and Bleeding Events During Palliative Care Stay

Outcome and population	HR for patients with any AC use after admission vs. patients with AC stopped at admission	HR while on AC (time-varying) vs. after stopping AC	
	HR crude (95% CI)	HR crude (95% CI)	HR adj^a^ (95% CI)
Suspected VTE			
All	0.66 (0.18–2.47)	0.31 (0.04–2.46)	0.25 (0.03–2.19)
Cancer patients	0.62 (0.17–2.30)	0.28 (0.04–2.22)	0.23 (0.03–1.95)
Bleeding event			
All	1.44 (0.54–3.85)	1.72 (0.58–5.15)	1.94 (0.61–6.15)
Cancer patients	1.35 (0.51–3.60)	1.54 (0.52–4.61)	1.93 (0.60–6.24)

^a^ Adjusted for patient history of thromboembolism and bleeding events.

CI, confidence interval; HR, hazard ratio.

When considering AC use as a time-varying exposure, the associations were larger between AC use and both events, with an HR of 0.31 for suspected VTE and an HR of 1.72 for bleeding, but still did not reach statistical significance. The association between AC use and the risk of VTE and bleeding was also larger after adjustment for patient history of these outcomes, suggesting that there could be confounding by indication of the crude HR for the AC effect.

### Death <72 hours following an event

The risk of death within 72 hours after a suspected VTE was 77% (95% CI 56%–91%) (Table 4) and 27% (95% CI 13%–46%) after a bleeding event. In cancer patients, the risk of death within 72 hours after a suspected VTE was 80% (CI 59%–93%) and 30% (CI 15%–49%) after a bleeding event. Very few non-cancer patients had a suspected DVT (one patient) or a bleeding event (three patients), and none died within 72 hours.

**Table 4.** Risk of Death Occurrence Within 72 Hours After a Venous Thromboembolism or a Bleeding Event

Event	Population	*n*/*N*	%	(95% CI)
Suspected VTE	All	20/26	77	(56%–91%)
	Cancer patients	20/25	80	(59%–93%)
	Non-cancer patients	0/1	0	(0%–98%)
Haemorrhagic event	All	9/33	27	(13%–46%)
	Cancer patients	9/30	30	(15%–49%)
	Non-cancer patients	0/3	0	(0%–71%)

## Discussion

This study showed that physicians decided to continue the administration of AC in one-third of the patients (35%) after their admission to a palliative care hospice and the majority of these (54%) had a history of VTE before admission. The prevalence of AC varies across institutions and countries. Holmes et al^12^ showed that 9% of lung cancer patients receiving hospice care were prescribed AC, Legault et al^13^ had around 13% of hospice patients continued AC at admission, whereas Kowalewska et al^14^ showed that 6.7% of all patients and 4.6% of cancer patients transferred to hospice care were prescribed AC therapy.

This supports the large practice diversity among physicians due to the absence of strong decision-making guidelines^15^ and evidence. Thus, physicians are left to rely on their own professional experience and the patient's medical history to support their ensuing decisions.

Most patients who continued taking oral AC stopped it during their stay (78%) mainly due to inability to swallow medication after disease progression.

As expected, we observed that the probability of developing a suspected VTE was lower in the patient group who continued taking AC (4.6%) whereas the probability of bleeding was higher (10.8%). This supports the Tardy et al^7^ study in which bleeding occurred in 11.0% of patients on AC, which was determined to be a significant independent risk factor for bleeding.

However, these findings should be treated with caution in view of the limited sample size. Although we observed potentially clinically important associations between AC use and the risk of VTE and bleeding events, the occurrence of VTE or bleeding events was rare, leading to low statistical power and large confidence intervals. A larger sample size than the one used in this study would be needed to exclude the possibility of chance findings.

Legault et al^13^ and Chambers^16^ studies showed that AC therapy was discontinued in most patients with advanced progressive disease without significant increase of the incidence of symptomatic VTE, which led them to challenge the routine use of AC in end-of-life patients. In this study however, we observed that for cancer patients experiencing a suspected VTE, the risk of death occurrence within 72 hours after the event was 80% and only 30% after a bleeding event.

Although the low incidence of death after a bleeding event in this population may be attributed to the mild nature of the majority of the observed bleeding events, in terms of survivorship, these findings suggest that it might be more advantageous to continue the administration of AC after admission in hospice. The higher incidence of death after a suspected VTE, although the strict causality cannot be established, and using medications that may extend survival is not always the patient's or family's priority in an end-of-life palliative care hospice, it does raise the possibility that AC may prevent a symptomatic or distressing death.

It is also of interest to mention that larger studies in the palliative population have shown no survival benefit to anticoagulation in the last year of life.^17^

More importantly, the fundamental objective remains to assess the symptomatic benefit of AC on palliative patients. One of the strengths of this study could be the observation of harms and benefits of AC based on the occurrence of symptomatic events observed when using clinical observation, which is the main tool at the clinician's disposal in the palliative care hospice environment.

These study results provide information on the clinicians' decisions at admission to a palliative care residence with regards to stopping or continuing the administration of AC. It also shows the possible advantage of continuing the administration of AC after admission to reduce the occurrence of VTE and the related death incidence. These data go in the same direction and support the known effects of AC use in cancer patients. Lastly, the results advocate for individualized patient-tailored decision making by clinicians conditional on the patient's history.

## Limitations

This study had some limitations. First, this was a retrospective study based on clinical observations made by treating physicians. In such end-of-life residences, there are no radiological imaging or laboratory tests done to support the clinical findings. Therefore, the absence of radiologic investigations requires that the prevalence of VTE, based on clinical observations only, be interpreted with caution.

Second, with the incidence of VTE and bleeding events being very low in general, the study had low statistical power to show statistical significance. However, the results are suggestive that there may be a clinically important effect of AC use at end of life that would be worth confirming in a larger study.

Finally, the sample size was not large enough to perform an analysis stratified by cancer site, as thrombosis was a rare event and only a few dozen cases of each cancer were reported. The results would not be significant or interpretable, because there would be very few expected cases of VTE for each site.

## Conclusion

Although many cancer patients admitted to hospice settings are already under AC, there is a lack of useful data and tools as well as clear formal guidelines for physicians with regards to anticoagulation strategies in end-of-life patients admitted in hospices. Using clinical data, this study provides additional insight into current physician practices, the utility and appropriateness of TPX strategies: incidence of suspected VTE and bleeding events, and the risks associated with continuing or discontinuing AC for patients with cancer nearing end of life.

Such data may assist in making and informing individualized care decisions. These findings suggest that it might be more advantageous to continue the administration of AC after admission in hospice. The higher incidence of death after a suspected VTE, although strict causality cannot be established, does raise the possibility that AC may prevent a symptomatic or distressing death.

Given the low incidence of events, larger appropriately powered prospective randomized studies focused on specific anticoagulation indications, although challenging, will be necessary to form the basis for guidelines.

## Abbreviations Used

AC: anticoagulotherapy
CI: confidence interval
DVT: deep vein thrombosis
HR: hazard ratio
IQR: interquartile range
TPX: thromboprophylaxis
VTE: venous thromboembolism
